# What *is* a prevention visit? A qualitative study of a structured approach to prevention and screening – the BETTER WISE project

**DOI:** 10.1186/s12875-021-01503-y

**Published:** 2021-07-19

**Authors:** N. Sopcak, C. Fernandes, M. A. O’Brien, D. Ofosu, M. Wong, T. Wong, M. Kebbe, D. Manca

**Affiliations:** 1 Department of Family Medicine, University of Alberta, Edmonton, Canada; 2 Department of Family and Community Medicine, University of Toronto, Toronto, Canada; 3 Strategic Clinical Networks, Alberta Health Services, Calgary, Canada; 4 Pennington Biomedical Research Center, Louisiana State University, Baton Rouge, USA

**Keywords:** Primary Prevention, Chronic Disease, Primary Care, Patient Care Team, Qualitative Research

## Abstract

**Background:**

This qualitative study is a sub-component of BETTER WISE, a comprehensive and structured approach that proactively addresses chronic disease prevention, screening, and cancer survivorship, including screening for poverty and addressing lifestyle risks for patients aged 40 to 65. Patients (*n* = 527) from 13 primary care clinics (urban, rural, and remote) in Alberta, Ontario, and Newfoundland & Labrador, Canada agreed to participate in the study and were invited to a one-hour prevention visit delivered by a Prevention Practitioner (PP) as part of BETTER WISE. We identified the key components of a BETTER WISE prevention visit based on patients’ and primary care providers’ perspectives.

**Methods:**

Primary care providers (PPs, physicians and their staff) participated in 14 focus groups and 19 key informant interviews to share their perspectives on the BETTER WISE project. Of 527 patients who agreed to participate in the study and were invited for a BETTER WISE prevention visit with a PP, we received 356 patient feedback forms. We also collected field notes and memos and employed thematic analysis using a constant comparative method focusing on the BETTER WISE prevention visit.

**Results:**

We identified four key themes related to a BETTER WISE prevention visit: 1) Creating a safe environment and building trust with patients: PPs provided sufficient time and a safe space for patients to share what was important to them, including their concerns related to poverty, alcohol consumption, and mental health, topics that were often not shared with physicians; 2) Providing personalized health education: PPs used the BETTER WISE tools to provide patients with a personalized overview of their health status and eligible screening; 3) Non-judgmental empowering of patients: Instead of directing patients on what to do, PPs evoked patients’ preferences and helped them to set goals (if desired); and 4) Integrating care for patients: PPs clarified information from patients’ charts and surveys with physicians and helped patients to navigate resources within and outside of the primary care team.

**Conclusions:**

The results of this study underscore the importance of personalized, trusting, non-judgmental, and integrated relationships between primary care providers and patients to effectively address chronic disease prevention, screening, and cancer survivorship as demonstrated by the BETTER WISE prevention visits.

**Trial registration:**

This qualitative study is a sub-component of the BETTER WISE pragmatic, cRCT, trial registration ISRCTN21333761 (date of registration 19/12/2016)

## Background

Chronic diseases such as heart disease, diabetes, and cancer account for almost 70% of all deaths worldwide [31]. While chronic disease prevention and screening (CDPS) typically takes place in the primary care setting, primary care physicians may not have the time or focus for CDPS, as they are already dealing with a broad array of complex issues including managing multiple chronic diseases, referrals, consult notes, and results from investigations completed in other settings [24]. Moreover, some research suggests that general health checks are not beneficial [15] and that the traditional annual physical exam offers only minimal benefits [23]. In Canada, an annual physical examination is non-standardized and may be conducted as part of a general health check-up for prevention and screening, although its merits have been debated [28]. Mehrotra and Prochazka [20], for instance, proposed that the number of annual physicals (administered by a physician) should be reduced in order to tackle prevention more effectively through health coaching.

One program that has widely demonstrated its effectiveness in improving CDPS outcomes is the BETTER (Building on Existing Tools to Improve Chronic Disease Prevention and Screening in Primary Care) Program [1, 11, 27]. BETTER is an evidence-based, integrated and comprehensive approach to CDPS in primary care. The intervention is administered by a healthcare professional within the primary care practice setting (e.g., registered nurse, licensed practical nurse, dietitian, kinesiologist), called a Prevention Practitioner (PP), who receives training in the BETTER approach and the BETTER tools [18, 19]. PPs meet with patients one-on-one for a one-hour prevention visit that focuses on primary prevention and screening of cancer, diabetes and heart disease and associated lifestyle factors such as diet, physical activity, smoking and alcohol [27]. The additional focus on modifiable lifestyle is important, since physical inactivity, unhealthy diets, smoking and alcohol are four major risk factors associated with increasing rates of chronic diseases [31]. BETTER has been successful with various populations and in different contexts. First demonstrated in a pragmatic cluster randomized controlled trial (RCT), the BETTER investigators found that a one-hour visit with a PP significantly improved CDPS outcomes in urban, well-resourced areas [11]. These findings were replicated in a subsequent implementation study, BETTER 2, which involved underserved, rural and remote settings in Newfoundland & Labrador, and provided further evidence that BETTER is “an effective approach to prevention in the real-world setting” ([1], p. 5). BETTER 2 also demonstrated that the BETTER approach was perceived to work well, could be successfully implemented in the primary care context [25], and was popular with patients who requested these types of visits as part of their regular primary care [26]. Furthermore, the BETTER approach has been adapted to community and public health settings [21], implemented as a government initiative in Newfoundland & Labrador, Canada (Government of Newfoundland and Labrador, [8]), and expanded by the BETTER Institute, which offers training and implementation support across Canada, including to First Nations, Inuit and Métis communities [3].

The BETTER WISE (Building on Existing Tools to Improve Cancer and Chronic Disease Prevention and Screening in Primary Care for Wellness of Cancer Survivors and Patients) project is a pragmatic, cluster randomized controlled trial (cRCT), embedded in a mixed method design with a qualitative evaluation and an economic assessment [17]. BETTER WISE extends the focus of the BETTER approach, to include not only primary prevention and screening of chronic disease, but cancer surveillance and screening for poverty. The aim of BETTER WISE is to determine if patients aged 40 to 65, including patients with and without a cancer history (breast, colorectal, and/or prostate), randomized to receive an individualized prevention visit with a PP improve cancer surveillance and prevention and screening outcomes. The primary outcomes are measured at the patient level and determined by a composite index, as compared to standard care in a wait-list control group twelve months after the initial prevention visit [17]. The aim of this sub-study is to identify the key components of a BETTER WISE prevention visit (conducted by a PP) based on the perspectives of patients and primary care providers (PPs, physicians and their staff).

## Methods

### Study setting

This qualitative study is a sub-component of the BETTER WISE cRCT, described in detail elsewhere [17]. The BETTER WISE team recruited 13 primary care clinics (urban, rural, and remote) in Alberta, Ontario, and Newfoundland & Labrador, Canada. Each clinic chose a healthcare professional to take on the role of PP for their setting. The PPs were typically primary care providers of an existing team and included three registered nurses, five licensed practical nurses, one registered dietitian, one pharmacist, one clinical medical assistant, one kinesiologist, and one clinic coordinator/manager. All PPs were trained to deliver the intervention using the BETTER WISE approach and the BETTER WISE toolkit [17], which included:

- Blended care pathways for primary prevention and screening of chronic disease, including behavioural lifestyle risk factors, as well as cancer surveillance for breast, colorectal, and prostate cancer survivors;
- Screening for poverty, informed by a tool developed by the Centre for Effective Practice for use in diverse primary care settings in Canada [4],
- Visual representations of the care maps (referred to as the “Bubble Diagrams”), which can be used directly with patients as a teaching tool and to assist with agenda setting;
- A patient health survey to capture a detailed prevention and screening history, confidence and readiness to change lifestyle habits, and for cancer survivors, a detailed cancer surveillance history; and
- Prevention Prescription and Cancer Surveillance Prescription templates that summarize the patient’s health status. These prescriptions provide patients and clinicians with a personalized plan aimed at facilitating shared decision making and enabling patients to actively engage in their health.

As part of a one-hour prevention visit, the PP reviewed with the patient the findings from the self-completed health survey and the medical chart (e.g. health status, family history, and lifestyle behaviours). PPs were trained to use an open, non-judgmental communication style to inform patients about their personal health risks and available screening using the BETTER WISE approach and tools [17]. PPs were also trained and certified in Brief Action Planning [12, 22], which “is grounded in the principles and practice of Motivational Interviewing and behaviour change theory and research, emphasizing compassion, acceptance, partnership and evocation” [22], in order to guide patients towards healthy lifestyle modification and behaviour change, and collaboratively set S.M.A.R.T. (specific, measurable, attainable, realistic, time-based) lifestyle or screening-related goals [17].

### Participants and recruitment

At each of the 13 participating primary care clinic sites, a list of all eligible patients 40 to 65 years of age was generated. Detailed eligibility criteria and recruitment strategies for the BETTER WISE cRCT have been published elsewhere [17]. For the qualitative sub-component, we invited all members of the 13 primary care clinics (including physicians, PPs, allied health professionals, administrators, and managers) to participate in focus groups or one-on-one interviews to share their perspectives on BETTER WISE. Focus groups were conducted in-person and written informed consent was obtained from all participants at the time of the focus group. Key informant one-on-one interviews were conducted over the telephone and written informed consent was obtained via e-mail prior to the interview. To enable patients to provide feedback with minimal time commitment, patients were invited to provide anonymous feedback using a short feedback form. Following their prevention visit with their PP, patients received an information letter along with the feedback form, which informed them that by completing the feedback form and submitting it to the team they were providing implied consent to participate in the qualitative component of the project.

### Data collection

First, we conducted the focus groups to facilitate meeting clinic team members in person, allow teams to get familiarized with the project, and to capture group thinking at each clinic setting. We then followed up with key informant interviews, which provided more in-depth conversations about BETTER WISE at each clinic. At least two members of the BETTER WISE team were present at each focus group: the qualitative research lead (NS), who was leading the conversation; and the research coordinator responsible for overseeing the project in that province, who took notes and shared observations during a debrief with the qualitative research lead after each focus group. One-on-one interviews were conducted by the qualitative research lead (NS) only. A semi-structured interview guide was used that explored each clinic’s context and processes as well as the impact of implementing BETTER WISE, including prevention visits by PPs and possible barriers and facilitators to the approach.

All focus groups and key informant interviews were audio recorded and transcribed verbatim (transcriptions were proofread and edited by NS and DO). Field notes and memos (descriptive, conceptual, or theoretical records of the data or the research process) were also collected. Patients were invited to complete a short, paper-based feedback form after their prevention visit with their PP (approximately 10 min to complete). Patients were asked about demographic details, expectations from the prevention visit, how the prevention visit met (or did not meet) their expectations, what they liked about their visit with their PP, what they would have liked to be different, and other comments. Providing feedback was completely voluntary and anonymous—patients could submit their feedback using a closed box located in their clinic waiting area or by mailing it to the project team by using a pre-addressed, stamped envelope.

### Data analysis

Data analysis started with the qualitative research lead (NS) listening to all interviews and focus groups, reading all transcripts and memos and coding each paragraph (i.e., assigning meaning) in a first round of coding. The thematic analysis used the constant comparison method informed by grounded theory [9, 10], comparing and contrasting emerging codes and themes from the data with other transcripts and tentatively conceptualizing the data in major themes as pertinent to the prevention visit. Based on recurring questions and misunderstandings from primary care providers regarding the prevention visit, we identified a knowledge gap about what the key components of a BETTER WISE prevention visit are. Thus, in a second round of coding, we reviewed the data (including memos and field notes) focusing on the prevention visit and key elements of the visit itself that were emerging. Patient responses were collected in REDCap®, an electronic data capture tool hosted and supported by the Women and Children’s Health Research Institute at the University of Alberta, then managed using a generated Excel spreadsheet (Microsoft® Excel, Version 16.20.) Two investigators (MK, NS) analyzed the patient data independently and created and refined emerging themes. In a third round of coding, the emerging themes from the patient data were integrated into the overall data set using the constant comparative method and reviewed and revised by the larger research team. These themes were then discussed and refined during several team meetings until consensus was reached. The large number of feedback forms received from patients (*n* = 356) and participants in focus groups and key informant interviews (*n *= 124) from different primary care settings enabled data saturation, as the emerging themes were validated by both patient feedback and primary care providers’ perspectives and no new relevant data emerged.

### Rigor of study methods

To ensure rigor, we used triangulation by involving diverse participants as data sources (physicians, clinic managers, PPs, allied healthcare staff, patients), different data collection strategies (focus groups, key informant interviews, patient feedback forms), and diverse settings (urban, rural, remote) in different provinces (Alberta, Ontario, Newfoundland & Labrador). Furthermore, memos and field notes (descriptive, conceptual, and theoretical records of the data or the research process) were also used to enhance and deepen the analysis. All authors participated in the data analysis. Multiple investigators met on several occasions to review the data, discuss, and refine emerging themes and resolve any discrepancies.

## Results

We conducted 14 focus groups with 124 individuals from the 13 participating primary care settings (16% male, 84% female; 54% from Alberta [AB], 30% from Ontario [ON], and 16% from Newfoundland & Labrador [NL]). Nineteen key informant interviews were conducted by telephone with 17 healthcare providers, including two men and 15 women. Two PPs left their position shortly after the study began and a second interview was completed after their departure. The key informants included 14 PPs, two physicians, and one clinic director, with seven individuals from AB, five from ON, and five from NL. See Table 1 for focus group and key informant interview participant characteristics.

**Table 1:** Selected characteristics of study participants involved in 14 focus groups and 19 one-on-one key informant interviews

**Total # of participants (focus groups)**	***N* = 124**
Characteristic	No. (%)
Gender	
Male	20 (16%)
Female	104 (84%)
Profession	
Primary Care Physician	38 (31%)
Admin/MOA/clerical staff	29 (23%)
Registered Nurse	16 (13%)
Clinic manager / coordinator / director	14 (11%)
Other clinicians (social worker, pharmacist, dieticians)	10 (8%)
Licensed Practical Nurse / Registered Practical Nurse	9 (7%)
Nurse Practitioner / Physician Assistant	6 (5%)
Family Medicine Residents	2 (2%)
Province	
Alberta	67 (54%)
Ontario	37 (30%)
Newfoundland & Labrador	20 (16%)
**Total # of participants (key informant interviews)**	***N*** = **17**
(2 PPs completed a second interview when they left role)	
Characteristic	
Gender	
Male	2
Female	15
Profession	
Registered Nurse	3
Licensed or Registered Practical Nurse	5
Physician	2
Nurse practitioner	1
Dietician	1
Pharmacist	1
Clinic director	1
Clinic coordinator	1
Clinic medical assistant	1
Kinesiologist	1
Province	
Alberta	7
Ontario	5
Newfoundland & Labrador	5

All Prevention Practitioners participated in both focus groups and key informant interviews

Of 807 patients invited to participate in the study, 527 patients agreed to a BETTER WISE prevention visit. 356 patients (39% male, 61% female; 61.8% from AB, 20.6% from ON, 17.6% from NL) returned feedback forms after their visit with a PP. We identified four key themes to a BETTER WISE prevention visit: 1) Creating a safe environment and building trust with patients: PPs provided sufficient time and a safe space for patients to share what was important to them including their concerns related to poverty, alcohol consumption, and mental health that were often not shared with physicians; 2) Providing personalized health education: PPs used the BETTER WISE tools (e.g., bubble diagram, prevention prescription) to provide patients with a personalized overview of their health status and eligible screening; 3) Non-judgmental empowering of patients: Instead of directing patients what to do, PPs evoked patients’ preferences and helped them to set goals (if desired); and 4) Integrating care for patients: PPs clarified information from patients’ charts and surveys with the physician and helped patients navigate resources within and outside of the primary care team.

### Theme 1: Creating a safe environment and building trust with patients

The first theme that emerged was the importance of creating a safe environment and PPs’ ability to build trust with patients. PPs and patients commented on the importance of having enough time and not feeling rushed (BETTER WISE prevention visits had 60 min allocated, including the study consent process). PPs created a relaxed atmosphere and a safe environment where patients felt listened to and cared for.

*It does take a time investment to do this properly. (…) The one thing about not being rushed is that first of all it allows for not just disclosures but it allows for people to feel comfortable like they are important and that their stories are important. (…) And it allows people the chance to explore their lives without being rushed out the door. Which is not something that we can always offer. [PP, KI008, AB]*

Patients commented on not feeling rushed and liked that they felt listened to by their PPs. Patients also commented on how they could address their concerns openly with their PPs and felt encouraged by the prevention visit. Patients stated feeling at ease in discussing their health due to particular visit characteristics, such as its confidentiality and open and relaxed nature.

*- [PP] took the time to listen to what was going on in my life and I didn’t feel rushed. [PP] recognized that mental health is just as important as physical health [Patient, female, AB]*

*- Pleasant, non-judgmental personality. No rush, patience, encouragement. Attainable suggestions [Patient, female, ON]*

*- How open I was. Able to talk about my health issues and the information about myself that I did not realize might be an issue causing some of the problems going on in my life [Patient, male, AB]*

In this safe and trusting environment, patients opened up to their PPs during their visit about their concerns such as poverty, alcohol consumption, and mental health. These were often not shared with their physicians due to concerns over how they may be viewed, not wanting to “waste” the physicians’ time, or not wanting to disappoint the physician.

*We uncover things that even the physician, their primary care provider for years was not even aware of. It just speaks to how valuable it has to be to give the patient the time and to be heard and to explore what they feel is most important to them as opposed to, you know, just the tests that need to be done or just the results that need to be conveyed or just the routine sort of follow-up that is sometimes done in their general care. [PP, KI 012, ON]*

*It’s totally different from the survey they filled out for me and what they’re actually telling me and when I actually met with patients, almost everybody has said: ‘I haven’t wanted to let my doctor down’. So, they haven’t been telling the truth. And specifically, regarding like the alcohol, how much physical activity, how much pain they’re in, (…) they felt like the doctor’s time was too valuable and they didn’t want to disappoint the doctor. [PP, KI007, AB]*

### Theme 2: Providing personalized health education

The second theme that emerged highlighted the importance of educating patients on their health in a personalized way. PPs used the BETTER WISE tools (e.g., bubble diagram, prevention prescription) to provide patients with a personalized overview of their: 1) health status, including mental health; 2) risk for cancer, diabetes, and heart disease and eligible screening; and 3) lifestyle behaviour (diet, physical activity, alcohol, smoking) and how it could affect risk for chronic disease or cancer recurrence. PPs commented on the importance for patients to have the information available and the opportunity to have a conversation about individual risks and benefits to promote informed choices.

*[Patients] don’t realize the benefits in having a FIT [fecal immunochemical test] (…) the people that I have met are not aware of the benefits of having the screening done. And I’m finding that I’m spending so much time on education and just talking to them about why. If you don’t want to have this done, it’s 100% the patient’s choice to decline treatment. But they need to be aware of what the benefits are and what the risks are of not having it or having it done. And I don’t think the docs just don’t have the time to do that because they’re trying to see so many patients. [PP, KI007, AB]*

Patients also expressed appreciation that the visit was personalized and informative, providing education on what tests were recommended for them instead of providing general health information.

*- Seeing my risk factors and actual condition on one sheet. Discussing how we will monitor the risk [Patient, female, ON]*

*- Each visit prompts me to discuss and reassess my goals and keeps me up to date with when I need to have screening tests done [Patient, female, NL]*

When patients had pre-existing conditions (that would be out of scope for BETTER WISE), PPs ensured that patients were followed up by their respective care provider for management. PPs also observed that some people had fallen “through the cracks”, as they did not get their screening completed.

*I think some of the things that stood out for me the most is patients that were in their earlier or mid 50s that have either have never gone for bloodwork, have never had their cancer screening done or even just some of those patients that just sometimes just end up falling through the cracks. [PP, KI 015, ON]*

PPs commented that a personalized prevention visit helped identify issues and initiate behaviour change, particularly regarding diabetes or pre-diabetes.

*We've had a few instances in which we've diagnosed diabetes. Also, some people who have quit smoking. Some people have devised exercise plans which they followed through with. Which is huge, from my perspective anyway. [PP, KI 008, AB]*

*We’ve caught actually three or four people [who] have been diagnosed with pre-diabetes just from BETTER WISE. I have started on actions to prevent diabetes. Catching it before it turns to diabetes. [PP, KI 014, ON]*

PPs also observed that patients were not always aware of their risk factors (based on family history or pre-existing conditions) or were not familiar with the guidelines or their personal next steps for prevention and screening in their age category. Patients appreciated this information, as it provided them the opportunity to play a more active role in their own health care.

*A lot of patients are coming in and they had a PAP done, say in January. They came in to see their doctor for the results in February but then no one ever told them what the next step was, like, ‘so in how many years do I need it, what’s the recommendation? (…) The feedback I have gotten is this that [the program] is very proactive because they feel that they’re actually part of their healthcare team, not just the healthcare team looking at them. And that seems to be what people want is they want to be part of the health circles that they know, that they’re proactive and they know what’s required for them for care. [PP, KI007, AB]*

### Theme 3: Non-judgmental empowering of patients

After reviewing their overall health status with the patient and learning more about their context, PPs asked patients if there was anything that they would like to do for their health. PPs were trained to both identify areas of improvement and areas of strength (e.g., reinforcing patients’ good habits). Instead of pushing patients to follow medical advice or change a certain behaviour, PPs operated from a strengths-based perspective and encouraged patients when they did not come up with an idea or had not completed their screening and/or their goals.

*Celebrating the good parts of people. (…) I think it’s common for us as human beings to feel like we’re not improving enough. So, this way we have a prevention project which doesn’t reinforce that feeling. That you are enough. And if you would like to make a plan as you are to move forward, that’s okay, too. [PP, KI 008, AB]*

*[This approach] doesn’t impose my ideas and it really helps these people come up with their own idea that they’re less likely to rebel against suggestions and I guess that really does increase compliance (…) The goal setting for me seems to be the biggest impact. [PP, KI 003, AB]*

Instead of directing patients on what to do, PPs evoked patients’ preferences about CDPS and helped them to set S.M.A.R.T. goals (if desired). PPs did this by asking open-ended questions, clarifying information, and by giving patients different options (including the option not to make a goal or plan).

*I said, ‘Look, this is no problem. The next time we meet in six months, if you think about something we can set a goal there or if there’s something that you think about later on in a couple of weeks or a month, just give me a call and we can set the goal over the phone.’ So, I’ve really kind of left it open ended that you know, ‘This is not something you have to do, but I am here to help you and support you’. [PP, KI 007, AB]*

*[If] they get stuck, I might say ‘can I make some suggestions that have worked for some other people in the past? Let’s make a menu of options and at the end say, maybe after hearing those things there’s something else that occurs to you?’ So, they still always have that option of adding their own idea into the mix. [PP, FG 001, AB]*

PPs emphasized that although patients sometimes had ideas about what goals they wanted to make, they needed some assistance with how to achieve their goals. Setting patients up for success by making small changes and talking it through was seen as one of the main benefits of a prevention visit as patients had the opportunity to think through different options.

*I’ve had some people even say when they started looking at their goals, they’re all ‘well I can’t go to the new center for swimming but I could probably go to the one on top of the road’. Like they kind of work that out themselves. And again, not a conversation that you’re going to have time to have with your physician. You need somebody to be able to talk to about that. [PP, KI 001, NL]*

Patients also commented on the goals as empowering. They commented on appreciating the goals being set in increments and the PPs working with them to make the goals realistic and achievable.

*- [PP] helped me make realistic goals [Patient, female, NL]*

*- Thank you! I have found having someone to develop prevention goals and checking in on my progress to be both rewarding and holds me accountable. [Patient, female, AB]*

*- Setting goals for my health with my prevention practitioner will go a long way for me. I feel like I’m doing this for myself but also have a coach motivating me in the background. (…) If I can maintain the goals I set for myself and confide in my practitioner to help me achieve good health, I will be very happy with myself. It could be a great mechanism for others who want to live healthier lifestyles as well. [Patient, male, ON]*

### Theme 4: Integrating care for patients

Integrating care for patients was the fourth theme that emerged from the data as a key component of the BETTER WISE prevention visit. While PPs had diverse backgrounds (e.g., dietician, pharmacist, nurse, etc.) and their own approach to relationship building with patients, BETTER WISE provided a structured approach aimed at integrating care for patients. Specifically, the prevention visit provided a space where a PP could catch patients out of date for screening, requiring follow-up on a pre-existing condition, or who shared concerns previously not expressed to their provider, such as mental health issues or screening positive for poverty. PPs could then connect patients to their primary care physician and/or other resources when needed and requested by the patient. This raised awareness for patients, providers, and other members of the primary care team, influencing the patient’s ongoing care.

*If [patients] haven't had their screening in so many years, whether it be a mammogram or a FIT test or whatever, when I send a note to the doctor, they say ‘Oh yeah, I haven't seen them in a while. That's, probably why’. But I think this is creating an awareness on both sides. You know, for the patient and for the physician [PP, KI 001, NL]*

Prevention visits provided an opportunity for PPs to help patients identify health concerns and share resources with patients that were available to them in their communities, such as specialists, dieticians, smoking cessation programs, and walking groups. Social determinants of health and mental health concerns were some of the issues that frequently arose and led PPs to connect patients to either their primary care provider or, where available, a social worker or counselling services. The BETTER WISE prevention visit assisted patients to become further connected with resources in their communities and encouraged them to take advantage of the healthcare team available to them.

*I had one gentleman he would come once a year and get a flu shot. And that was it. And he wouldn't come to the doctor for anything else. And he was very against screening, the fecal occult in particular. Not against it but just did not want to do it, right? So, he hadn't had bloodwork in a long time and he chose to go for some bloodwork after the BETTER WISE appointment. And he was diagnosed with prediabetes. So, he had come back in to have a visit with me as a family health team member for diabetes education. And during that visit he-, I explained to him the new FIT testing that just went live yesterday and now he wants to go, he wants to get that screening done now. So, without the BETTER WISE I don't think he would have ever done it. [PP, KI014, ON]*

*Maybe [the doctors] didn't know the full extent of sort of the challenges that the patients are facing. And so sometimes you uncover a little bit more in terms of how things are, how they're coping at home or sort of their family dynamics or if there's you know maybe they're they've been hesitant about seeking help but they didn't want to tell anyone about their mental health concerns. And so sometimes when you have built that relationship with the client then they actually feel comfortable opening up to you. And so, you have to expand, and it enables me to explore and ask permission ‘Is it OK if I share this with your primary care provider so that we can see what supports are out there for you’? [PP, KI 012, ON]*

Patients, in turn, appreciated having someone to help them stay on track with screening, encourage them to discuss health concerns with their family physician, and to learn about and be connected to available resources.

*- I appreciated the contact as I tend to ignore “timelines” – It made me think about where I am vis-à-vis health issues / prevention. The Prevention Practitioner told me about available resources which I found reassuring (and appreciated!) [Patient, female, AB]*

*- [PP] presented plans to improve my well-being and ensured a follow-up visit with my doctor, given my complex conditions. [Patient, male, AB]*

*- Covered a lot about health and what can be done to improve it. (…) It opened my eyes that there is help out there and I am not alone to get healthier [Patient, male, ON]*

Physicians who participated in the key informant interviews and focus groups also appreciated having the PPs as someone to update information in the patients’ charts and to alert them when patients needed their follow-up.

*[PP] will message me if she identifies something that needs to be done, so someone that needs to do screening or is requiring follow-up that hasn’t happened yet. So, it’s been positive in that it takes some of that brain pressure off of me to remember and also drives the visit for a specific focus on that. And I would even say that [PP] is picking things up that I might otherwise have missed, when there is sometimes an exception to the screening. [Physician, KI 017, ON]*

One of the physicians participating in BETTER WISE commented that the PP identified patients who had financial difficulties of making ends meet and of whom the physician was not aware.

*“In the seven people [PP] has done for me, already, she picked up on the poverty screen a couple that I had no idea (…) because there’s a space and time for when you’re asking the question and I would have never known to ask the question because they don’t tell you and you wouldn’t guess. And so actually that was tremendously helpful already because [PP] got them dialed into supports and has made a big difference” (Physician, FG 003, AB)*

## Discussion

Implementing prevention and screening in primary care has been identified as challenging due to primary care providers’ time restrictions and the added burden to stay on top of guideline evidence, while communicating with patients effectively and taking patients’ preferences and values into account [30]. In this qualitative study, we identified four key components of a prevention visit as exemplified by BETTER WISE project based on patients’, PPs’, and physicians’ perspectives.

The first theme, creating trusting relationships with patients highlights the significance for patient to feel they are in a safe environment where they are not rushed and feel listened to. While most primary care providers believe in the importance of listening skills, healthcare agendas and time limitations can easily get in the way of truly listening to patients’ concerns [6]. Trusting relationships between patients and primary care providers have been found to be an important factor in areas such as public health [5], family practice [29], as well as in previous iterations of BETTER [19, 26]. Bensing et al. [2], for instance, found that when patients can freely voice their preferences for interactions with primary care providers, “they tend to focus on ‘fostering the relationship’ with an emphasis on personal attention, warmth and empathy” (p. 287).

Providing personalized health education to patients emerged as second theme, indicating that patients found it important to get a comprehensive picture of their health status, including areas where they were doing well, and to be informed of areas where they can improve. This theme was also reflected in previous BETTER studies: individualized and personalized care was a key component in the first BETTER iteration [19] and the following implementation study, BETTER 2 [25, 26]. Primary care research suggests that healthcare communication needs to be personalized and patient-centred, as making changes in lifestyles and health behaviour is particularly difficult [14]. Patients in our sample attributed their positive experiences with the BETTER WISE prevention visit to their PP, particularly characteristics related to effective communication. In addition to positive patient experiences in healthcare, meta-analyses have shown that effective communication by a provider, which involves empathy, question-asking, and establishing rapport, can lead to meaningful improvements in patient adherence to care [32] and health outcomes (e.g., self-efficacy) [7].

Effective communication was also part of our third theme, non-judgmental empowerment of patients. Patients felt empowered and motivated to consider lifestyle changes and set realistic goals but also were not judged when they decided not to make a goal. These results are similar to Jansink et al. [13], who recommend a counseling-based approach in the context of lifestyle behaviour change, that includes empathy and listening to patients’ needs as they found that nurses get tired of advice giving, particularly when constantly repeating the same messages with low success rates in patient uptake.

Lastly, the last theme, integrating care for patients was seen as important by both patients and primary care providers. By clarifying information with primary care providers, updating medical charts, and linking patients with their primary care provider or other resources within or outside of the clinic setting, PPs were able to support continuity of care as team members. We found in the BETTER 2 implementation study that differences in primary care perspectives also differed based on whether they practiced *in* a team (i.e., a physician with helpers) versus *as* a team (a fully multidisciplinary team to improve the care provided to patients in a medical home model) [25]. The results of the present qualitative study fit with previous research, which suggests that “team-based care, systems of care that accommodate preventive services, and willingness of patients to seek out and engage in preventative care” are essential parts of a multipronged approach for increased preventive services ([16], p. 3). Further research is needed to determine how a PP could be made available and possibly adapted to other primary care settings considering financial and organizational implications.

### Strengths and limitations

Our study has both strengths and limitations. First, patients were enrolled in the BETTER WISE cRCT by invitation on a voluntary basis, which may represent a selective sample of the population with potentially different interests in prevention and motivation for change than patients who received our invitation but chose not to participate. Second, while our patient data in the form of open-ended feedback forms may not have been amenable to the in-depth, rich descriptions found in our focus groups and key informant interviews, we chose this method of data collection for feasibility and practicality purposes based on previous experience [26]. We did receive 356 feedback forms from 527 patients who agreed to participate in the study and were invited for a BETTER WISE prevention visit, indicating that the majority of patients (67.2%) welcomed the opportunity to share their perspectives on the visit.

## Conclusion

Primary care physicians are already dealing with a broad array of complexity issues, such as managing the burden of chronic disease [24]. Previous iterations of BETTER indicate that preventive visits with PPs improve cancer and chronic disease screening and prevention outcomes among patients due to the partnership approach that exists between the patient and the provider [1, 11, 19, 25]. Since PPs are well-trained to be champions of health promotion, they are in an ideal position to enhance accessibility to preventive health by providing the required expertise and resources for change. The results of this study address the gap of knowledge regarding the key components of a prevention visit, as valued by patients and primary care providers. Our results of this study underscore the importance of personalized, trusting, non-judgmental, and integrated relationships between primary care providers and patients in the context of chronic disease prevention and screening, such as the BETTER WISE prevention visits.

## Authors’ information

NS is the qualitative research lead for BETTER WISE. CF is the lead research coordinator for BETTER WISE, the lead coordinator for the BETTER Program, and executive director for the BETTER Prevention Practitioner Training Institute with expertise in qualitative and quantitative methodologies. MAO is an assistant professor and scientific associate with the Knowledge Translation Research Network with expertise in qualitative methodologies. DM is an active family physician and Director of Research of the Department of Family Medicine Research Program with expertise in qualitative and quantitative methodologies. MW is a study coordinator for BETTER WISE. DO is a research assistant for BETTER WISE. TW is a patient advisor for BETTER WISE. MK was involved as graduate research assistant (GRA) during BETTER WISE.

## Data Availability

The datasets generated or analysed during the current study are not publicly available due to concerns that participants could be identified. Patient data is available from the corresponding author on reasonable request.

## Abbreviations

AB: Alberta, Canada
BETTER: Building on existing tools to improve chronic disease prevention and screening in primary care
BETTER WISE: Building on exiting tools to improve cancer and chronic disease prevention and screening in primary care for wellness of cancer survivors and patients
CDPS: Chronic disease prevention and screening
cRCT: Cluster randomized controlled trial
FG: Focus group
FIT: Fecal immunochemical test (screening test for colon cancer)
KI: Key informant
NL: Newfoundland and Labrador, Canada
ON: Ontario, Canada
RCT: Randomized controlled trial
S.M.A.R.T.: Specific, measurable, attainable, realistic, time-based
PAP: Papanicolaou test (screening test for abnormal cells in cervix)
PP: Prevention Practitioner
